# Weak correlations between cerebellar tests

**DOI:** 10.1038/s41598-020-65886-1

**Published:** 2020-06-02

**Authors:** Karolina Löwgren, Rasmus Bååth, Anders Rasmussen

**Affiliations:** 1 Department of Clinical Sciences, Lund University, BMC F12, SE-221 84 Lund, Sweden; 2 Department of Philosophy, Cognitive Science, Lund University, SE-223 62 Lund, Sweden; 3 Department of Experimental Medical Science, Lund University, BMC F10, SE-221 84 Lund, Sweden; 4 Department of Neuroscience, Erasmus University Medical Center, 3000 DR Rotterdam, The Netherlands

**Keywords:** Classical conditioning, Developmental biology, Neurodevelopmental disorders

## Abstract

Eyeblink conditioning, finger tapping, and prism adaptation are three tasks that have been linked to the cerebellum. Previous research suggests that these tasks recruit distinct but partially overlapping parts of the cerebellum, as well as different extra-cerebellar networks. However, the relationships between the performances on these tasks remain unclear. Here we tested eyeblink conditioning, finger tapping, and prism adaptation in 42 children and 44 adults and estimated the degree of correlation between the performance measures. The results show that performance on all three tasks improves with age in typically developing school-aged children. However, the correlations between the performance measures of the different tasks were consistently weak and without any consistent directions. This reinforces the view that eyeblink conditioning, finger tapping, and prism adaptation rely on distinct mechanisms. Consequently, performance on these tasks cannot be used separately to assess a common cerebellar function or to make general conclusions about cerebellar dysfunction. However, together, these three behavioral tasks have the potential to contribute to a nuanced picture of human cerebellar functions during development.

## Introduction

In eyeblink conditioning, an initially neutral conditional stimulus (CS), often a tone, is repeatedly presented before an unconditional stimulus (US), often an air-puff to the cornea, which elicits a reflexive unconditional response (UR). Eventually, the CS acquires the ability to elicit a conditioned blink response (CR), that occurs before the US. In finger tapping, a subject is asked to follow a rhythmic stimulus, often auditory, and then reproduce the tempo after the auditory stimulus has ended. In prism adaptation, the subject wears wedge prisms that displace the visual field laterally, causing a pointing error that diminishes quickly with training.

Research on animals and humans shows that the cerebellum plays a critical role in the acquisition and expression of CRs during eyeblink conditioning^1–5^. In finger tapping, the cerebellum is recruited during the ongoing sensorimotor timing control^6^. The cerebellum’s importance for eyeblink conditioning and finger tapping is perhaps unsurprising given that both tasks require precise timing in the sub-second range – which is a key function of the cerebellum^7,8^. Indeed, based on their shared reliance on precise timing, it has been suggested that finger tapping and eyeblink conditioning depend on the same neural mechanisms^9^. Prism adaptation, by contrast, is thought to rely on cerebellar spatial processing^10,11^.

While there is ample evidence showing that performance in eyeblink conditioning, finger tapping, and prism adaptation all involve the cerebellum^12–14^, it is also clear from animal studies and fMRI studies on humans that these three tasks rely on partially separate cerebellar and extra-cerebellar neuroanatomical sites^15–17^. Eyeblink conditioning engages Larsell’s cortical lobule VI^18,19^. Studies using fMRI in humans show that finger tapping activates lobules IV, V, and VI^17,20^, suggesting a partial overlap with the structures engaged in eyeblink conditioning. Prism adaptation and reaching errors engage cerebellar lobules V, VI, and VII^21^, though to abolish prism adaptation in macaque monkeys requires substantial cerebellar lesions^10^. Eyeblink conditioning, finger tapping, and prism adaptation are all quantifiable, non-invasive, culturally neutral, and relatively simple to participate in. These tests have been used separately when investigating cerebellar dysfunction in patients with neurodevelopmental disorders^22^. However, apart from one study which showed that CR acquisition during eyeblink conditioning correlated with finger tapping variability in adults^23^, few studies have looked at correlations between performances on different cerebellar tasks. A test battery combining these tasks could shed more light on the cerebellar contribution to neurodevelopmental disorders associated with cerebellar dysfunction, such as attention deficit hyperactivity disorder (ADHD), schizophrenia, and autism spectrum disorder^24–27^. Behaviorally, these disorders have been linked to performance deficits on cerebellar dependent tasks^2,28,29^, although the results are sometimes inconsistent between studies^22^.

We have previously shown that performance in eyeblink conditioning is dependent on age^30^, and on the interstimulus interval (ISI)^31^. Studies on mice and ferrets often use an ISI around ~300 ms, to which animals learn consistently^32–34^. Humans, by contrast, learn poorly with a 300 ms ISI^31^. One possible explanation for this discrepancy is that humans rely more on voluntary, non-cerebellar, processes when trained in eyeblink conditioning^35^, though this effect may also depend on which ISI is used. As a result, the extent to which performance on eyeblink conditioning captures cerebellar function may depend on the ISI used, and if training with a particular ISI does not require the cerebellum, then we should not expect strong correlations with performance on other cerebellar tasks.

The purpose of this study was to investigate the relationships between eyeblink conditioning, finger tapping, and prism adaptation, in both school-age children and adults. Furthermore, to determine whether the degree of correlation depended on the ISI during eyeblink conditioning, the adults were trained either with a 300 or 500 ms ISI. Based on the evidence that eyeblink conditioning, finger tapping, and prism adaptation are associated with partially overlapping cerebellar neural activity, we hypothesized that performance on these tasks would correlate to some extent and thus reflect the common functioning of the cerebellum.

## Methods

### Subjects

Included in the study were 42 children (22 females and 20 males), age 6–11 years (mean = 8.8, SD = 1.3), recruited from elementary schools in middle-class socioeconomic areas in southern Sweden. From the original sample size of 46 participants (Löwgren *et al*.^30^), three were excluded due to technical issues during the eyeblink registrations, and one was excluded due to non-verbal intelligence quotient (IQ) below 65 (percentile 1) on Raven’s colored progressive matrices^36,37^. All children displayed normal hearing (screening level 20 dB HL with pure tone audiometry by the modified Hughson-Westlake method, ISO 8253-1, with GSI 66 screening audiometer), and their average IQ score was 105 (SD = 16, min = 80, max = 140). On average, 68% of the pupils in these schools had at least one parent with higher education. None of the children needed extra pedagogical support in school, were on any medication, nor had any eye disease. None of the children used contact lenses, although 6 reported vision deficits. Based on a questionnaire, 37 subjects were categorized as right-handed and 5 left-handed.

We also included 44 adults (26 females and 18 males), age 18–55 years (mean age = 27.9 years, SD = 8.2), recruited from the student population at Lund University. From the original sample size of 49 adult participants^31^, one was excluded due to very late reaction time responses (median = 364 ms) (method described below) and four due to technical issues during the eyeblink registrations. The adults displayed normal hearing (screening level 20 dB HL with pure tone audiometry by the modified Hughson-Westlake method, ISO 8253-1, with GSI 66 screening audiometer), 41 adults were right-handed and 3 left-handed. Ten of the adults reported that they were on medication (reported more than once: contraceptive pills, antihistamine, and asthma medication). None had any eye disease while 16 suffered from vision loss and 5 used contact lenses.

### Procedure

This study was approved by the Swedish Ethical Review Authority in Lund, Sweden (dnr 2009/383) and all procedure complied with the relevant guidelines and regulations. All subjects or their legal guardians signed a consent form before the testing. The testing was performed in a secluded room with low background noise (<45 dB (A) (L_eq._ 60 s), measured with Bruel & Kjær 2225 sound level meter). The children were tested in school on three different occasions (1. Finger tapping, 2. Eyeblink conditioning, and 3. Prism adaptation), with a week in-between. The adults were tested in the Humanities laboratory at Lund University in one single session. Prism adaptation was added to the test paradigm only for the last 19 of the children (mean age = 7.9, SD = 1.0, 11 females, 8 males). All the adults were tested on all three tests.

#### Eyeblink conditioning

Classical delay eyeblink conditioning was performed using equipment developed by Neurasmus, Rotterdam, The Netherlands. The equipment, as well as the procedure and data analysis, are described in detail by Löwgren *et al*.^30^. Briefly, the subjects watched a film while they received up to 10 blocks of 10 trials. To examine the full CR profile, the airpuff was sometimes left out. Such unpaired probe trials were presented mainly towards the end of the training (for details, see Löwgren *et al*.^30^). The conditional stimulus (CS), a 1 kHz tone, was presented binaurally through headphones during 300 or 500 ms with a sound pressure level of 68 decibels. Nineteen of the adults, 18–44 years old (mean age = 26.3, SD = 7.4, 9 females and 10 males), were tested with the shorter 300 ms interstimulus interval (ISI) between the onset of the CS and the unconditional stimulus (US). The remaining 25 adults, 20–55 years old (mean age = 29.1, SD = 8.8, 17 females and 8 males), were tested with the longer 500 ms ISI. All children were tested with 500 ms ISI. The US, a 15 ms air puff of 1 bar, was aimed at the left cornea from a distance of 1–2 cm. The intertrial interval (ITI) was programmed to vary pseudo-randomly between 15 and 25 seconds. Although it is commonplace to train humans for 60–100 trials in eyeblink conditioning, learning curves reveal that learning takes place primarily in the first 10–20 trials^30,31,38^. Therefore, to assess the performance during eyeblink conditioning, we used the percentage of conditioned responses (CRs) and the onset latencies in blocks 2–5: 40 trials, mainly containing paired trials.

#### Prism adaptation

Prism adaptation was tested with a pair of wedge prism glasses (Neurasmus), which shifted the visual field to the left. The subjects were instructed to rapidly point to a target on the wall using the dominant arm and index finger. To avoid real time corrections of the arm movement, based on visual information, the subjects were instructed to close their eyes before pointing at the target. They were informed to open their eyes immediately after every trial to get visual feedback on the actual location of the finger, hand, and arm. The test consisted of 15 (children) or 30 (adults) trials equally divided into: 1. Training before adaptation, without the prisms. 2. Adaptation to the prisms, with the prisms on. 3. Testing after the adaptation to the prisms, without the prisms. The subjects gradually adapted their motor response of the arm movement to the new sensory input due to the shift in the visual field and then gradually readapted after removing the prisms. To measure the magnitude of the adaptation, we used the deviation from the intended target on the first trial after having removed the prisms.

#### Finger tapping

The isochronous serial interval finger tapping was conducted using custom made software, developed by Guy Madison^39^. A short ~30 ms auditory ticking metronome sound, with an inter onset interval of 524 ms, was presented at 65 dB (A) (85 dB peak). The test started with a practice session consisting of 15 repetitions of the stimulus, to which the subjects synchronized their tapping, immediately followed by the self-paced production phase when the subjects continued to keep the beat by tapping 31 times without any guiding sounds. After this first practice came 4 measuring trials containing 15 synchronization and 70 continuation taps each. The subjects were instructed to tap short and distinctively on the spacebar using the index finger of their dominant hand. They were instructed to maintain the same exact tempo as the metronome sound. The adults were tested through Sennheiser HDA 200 headphones and the children were tested in a sound field setup, where the sound level was calibrated (by ISO-TECH SLM52N sound level meter) at the distance 0.5 m from the computer’s internal loudspeaker (HP Compaq dx2000 Microtower PC) in head height for the children. We looked at the mean of the standard deviation of the inter-response intervals from each trial to capture the timing ability of the participants during the self-paced production phase (Production SD). When analyzing the data, the first three taps were removed from each trial to exclude the transition from the synchronization phase to the production phase.

#### Reaction time

A simple auditory reaction response time (RT) test was performed in conjunction with the finger tapping. The participants were instructed to press the spacebar with the dominant hand’s index finger immediately after hearing a short auditory transient sound at 65 dB (A). The test consisted of 1×12 practicing trials followed by 1×30 measuring trials. Reaction time does not appear to be linked to cerebellar function^40^ and was included here to be able to detect outliers and potentially control for motor output speed^41^.

### Statistical analysis

Age, RT, and IQ measures were included in the analyses as subject variables. To estimate the correlation between the measured performance on each task we used Pearson’s correlation coefficient. To control for age in the child group, we fitted linear regression models for each performance measure with age as the predictor and the performance measure as the outcome variable. The correlation was then estimated between the residuals of the performance measures, that is, what was left after the effect of age had been taken into account. Correlations were estimated by Bayesian estimation using the brms statistical package^42^ and the R statistical environment^43^. We used the default non-informative priors supplied by brms and each correlation estimate was based upon 20,000 posterior samples from the model.

When describing the result, we give the magnitudes of the correlation estimates calculated as the mean posterior correlation. The uncertainties around these estimates are given as confidence intervals (CI) calculated as equal-tailed probability intervals. In the analysis, we have focused on the magnitudes of the correlation estimates. and not on the null hypothesis significance testing as it is implausible, a priori, that the population level correlations between the different performance measures are exactly zero. However, when analyzed using classical null hypothesis significance testing, none of the correlation estimates presented in the result section for the adult group, nor for the child group when controlling for age, were statistically significant at a 0.05 alpha level. We did not adjust for multiple comparisons since doing so would merely result in larger p-values, which would further strengthen our conclusions.

## Results

In eyeblink conditioning, the children produced 27 percent (SD = 25) conditioned responses (CRs), whereas the adults produced 43 percent (SD = 29) CRs, on average in blocks 2–5. The mean CR onset was 331 ms (SD = 69) for the children. Adults tested with an interstimulus interval (ISI) of 300 ms reached on average 35 percent CRs (SD = 30), and the mean CR onset latency was at 214 ms after the conditional stimulus (CS) onset (SD = 22), whereas the adults tested with an ISI of 500 ms reached 48 percent CRs (SD = 27) and showed mean CR onset at 316 ms (SD = 46). After prism adaptation, the children deviated 2.5 cm (SD = 2.2) from the target and the adults 3.9 cm (SD = 1.9) on average. During finger tapping, the mean standard deviation (Production SD) of the inter-response intervals was 45 ms (SD = 10) in the child group and 23 ms (SD = 6) in the adult group. The reaction response time (RT) resulted in a median of 328 ms (SD = 84) for the children and 191 ms (SD = 21) for the adults.

We calculated the correlations between the measures of eyeblink conditioning (‘Percent CR’, and ‘CR onset’ in ms after CS onset), self-paced finger tapping (‘Production SD’ in ms), and prism adaptation (‘Prism deviation’ in cm) in the group of school-aged children. ‘RT’ in ms, non-verbal standard ‘IQ’ score, and age in years were also included in the analysis. We applied the same correlation analyses to the adults’ performance measures but without any IQ measurement.

### Weak correlations and age effects among the children

**Figure 1:**
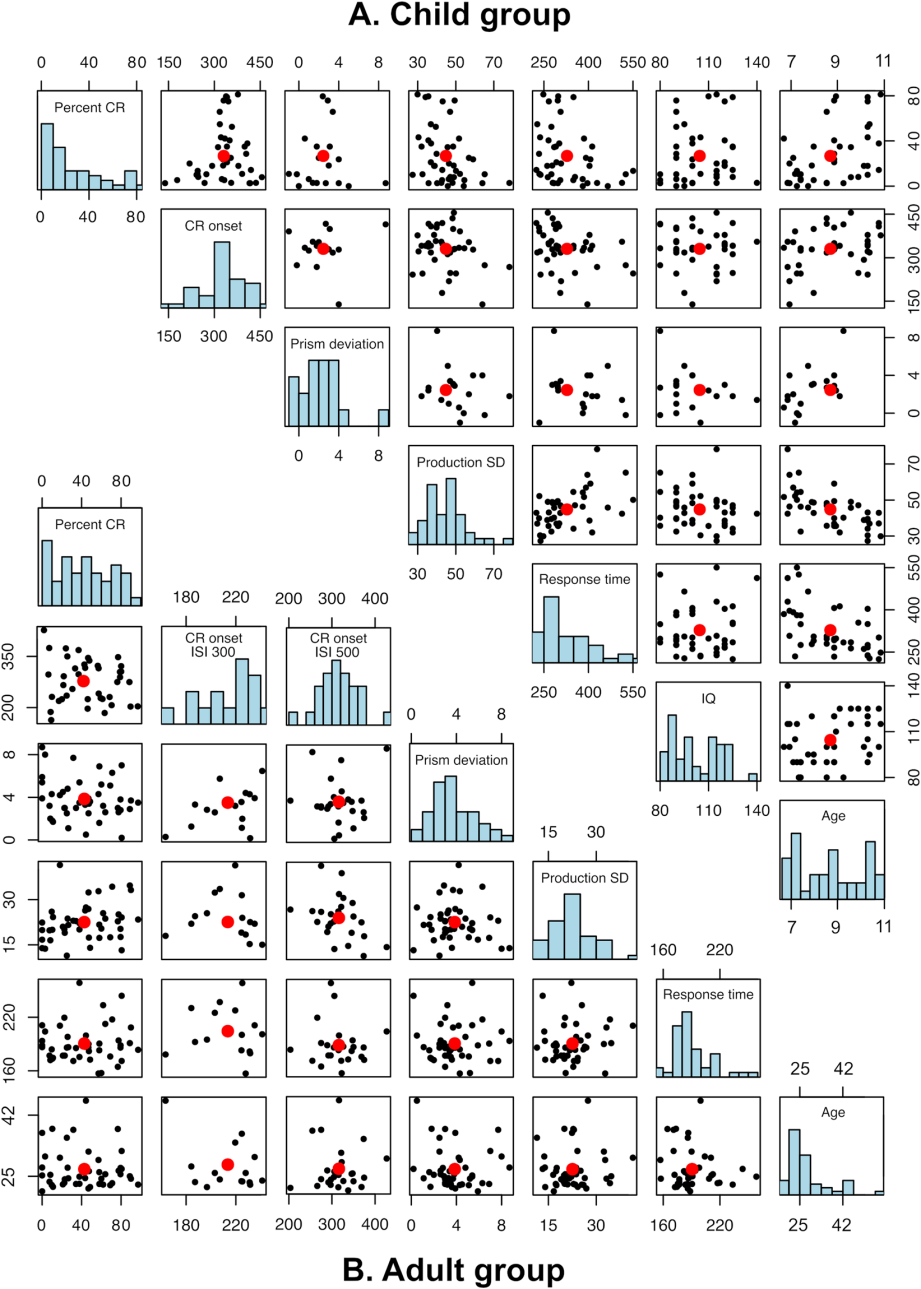
Scatter plot matrix. The distribution of the data for the child (A) and the adult (B) groups. Each black point represents an individual’s measurements and red points show the sample means of the measurements. The scales of each plot are in accordance with the units of the performance measures: ‘Percent CR’; ‘CR onset’ in ms after CS onset; ‘Prism deviation’ in cm from target at 0; ‘Production SD’ in ms; ‘Response time’ in ms; ‘IQ’ in standardized scores; ‘Age’ in years.

When plotting the pairwise distributions of the performance measures, no visually striking relationships appear (Fig. 1A). However, all the measures correlate moderately with age (Table 1), with an exception for IQ score, which is a standardized measure and adjusted to age norms. With higher age, the CR percentage increases; the CR onset approaches the US onset; the prism deviation increases (suggesting more adaptation); the tapping variability decreases; and, the RT shortens. When controlling for age among the children using a linear regression model, the correlations between the performance measures on the tests are small or non-existent (Table 2). The strongest correlation estimate is a weak negative correlation between CR percentage and prism deviation. But even that correlation estimate, like all other age-controlled estimates, has a 95% confidence interval (CI) that overlaps zero, indicating that there is no strong evidence that any of the correlations are substantial.

**Table 1:** Correlation table – children.

	Percent CR	CR onset	Prism dev.	Prod. SD	RT	IQ
Prism dev.	−0.01 [−0.45, 0.44] p = 0.972	0.08 [−0.37, 0.5] p = 0.704				
Prod. SD	−0.34 [−0.58, −0.06] p = 0.017	−0.21 [−0.48, 0.08] p = 0.146	−0.19 [−0.58, 0.24] p = 0.346			
RT	−0.3 [−0.55, −0.01] p = 0.038	−0.29 [−0.55, 0] p = 0.045	−0.17 [−0.58, 0.28] p = 0.408	0.47 [0.21, 0.68] p < 0.001		
IQ	0.12 [−0.18, 0.41] p = 0.399	−0.04 [−0.33, 0.26] p = 0.810	−0.19 [−0.56, 0.25] p = 0.362	−0.2 [−0.47, 0.1] p = 0.178	0.04 [−0.25, 0.33] p = 0.769	
Age	0.45 [0.18, 0.67] p = 0.002	0.32 [0.03, 0.58] p = 0.028	0.5 [0.1, 0.78] p = 0.013	−0.6 [−0.77, −0.37] p < 0.001	−0.52 [−0.71, −0.27] p < 0.001	0.24 [−0.06, 0.51] p = 0.094

Estimates of correlation coefficients between the different measures in the child group, together with 95% CIs, and p-values.

**Table 2:** Correlation table – children, controlling for age.

	Percent CR	CR onset	Prism dev.	Prod. SD	RT
Prism dev.	−0.33 [−0.7, 0.13] p = 0.129	−0.13 [−0.56, 0.33] p = 0.521			
Prod. SD	−0.09 [−0.39, 0.22] p = 0.543	−0.02 [−0.33, 0.29] p = 0.884	0.13 [−0.31, 0.54] p = 0.536		
RT	−0.08 [−0.37, 0.23] p = 0.606	−0.15 [−0.43, 0.15] p = 0.307	0.04 [−0.42, 0.48] p = 0.827	0.22 [−0.08, 0.5] p = 0.127	
IQ	0.01 [−0.29, 0.3] p = 0.947	−0.12 [−0.41, 0.18] p = 0.423	−0.12 [−0.54, 0.33] p = 0.559	−0.06 [−0.34, 0.25] p = 0.697	0.22 [−0.09, 0.49] p = 0.137

Estimates of correlation coefficients between the different measures when controlling for age in the child group, together with 95% CIs, and p-values.

### Weak correlations among the adults

**Figure 2:**
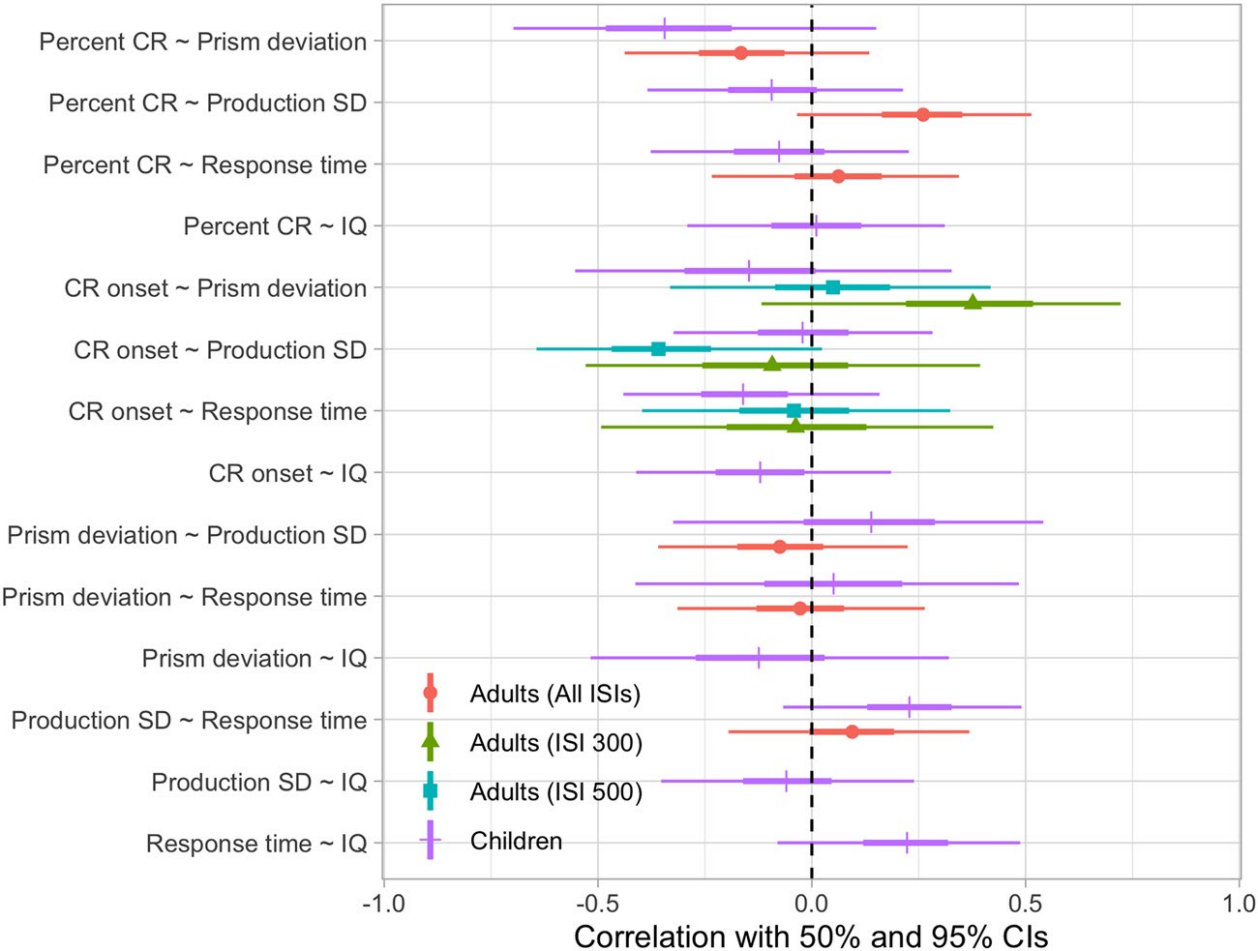
Correlation plot. The strength and direction of the correlation for the child and adult group. For the child group the correlation estimates when controlling for age are shown. The thicker line represents a 50% CI, while the thinner line represents a 95% CI, for each correlation. A negative correlation (to the left of 0.0), means that one of the performance measures increases when the other decreases. A positive correlation (to the right of 0.0), means that if one of the performance measures increases/decreases so does the other. The 95% CI overlaps 0.0 in all correlation estimates, and a 50% CI overlaps 0.0 in a majority of the correlation estimates. When interpreting this figure, note that a high score on some measures does not necessarily mean better performance. For example, a high score on finger tapping variability means that the subject could not maintain the rhythm.

As with the child data, the correlations between the measures in the adult group are weak (Table 3 and Fig. 1B). In addition, all estimated correlations have 95% CIs that cross the zero mark, meaning that the directions of the correlation estimates are uncertain (Fig. 2). We also separately analyzed the two adult groups, tested either with 300 or 500 ms ISIs during the eyeblink conditioning. Again, the correlations are generally weak, and the 95% CIs cross the zero mark (Table 4).

**Table 3:** Correlation table – adults.

	Percent CR	Prism dev.	Prod. SD
Prism dev.	−0.16 [−0.44, 0.14] p = 0.264		
Prod. SD	0.25 [−0.03, 0.51] p = 0.075	−0.07 [−0.36, 0.22] p = 0.619	
RT	0.06 [−0.23, 0.34] p = 0.671	−0.03 [−0.31, 0.26] p = 0.851	0.09 [−0.2, 0.36] p = 0.525

Estimates of correlation coefficients between the different measures the adult group, together with 95% CIs, and p-values.

**Table 4:** Correlation table – adults, split by eyeblink conditioning ISI.

	Percent CR		CR onset	
	ISI 300	ISI 500	ISI 300	ISI 500
Prism dev.	−0.07 [−0.49, 0.38] p = 0.754	−0.13 [−0.5, 0.27] p = 0.498	0.36 [−0.12, 0.72] p = 0.106	0.05 [−0.33, 0.42] p = 0.804
Prod. SD	0.27 [−0.17, 0.64] p = 0.188	0.34 [−0.04, 0.65] p = 0.062	−0.08 [−0.53, 0.39] p = 0.710	−0.35 [−0.64, 0.02] p = 0.053
RT	0.22 [−0.23, 0.6] p = 0.296	−0.01 [−0.37, 0.37] p = 0.969	−0.04 [−0.49, 0.42] p = 0.870	−0.04 [−0.4, 0.32] p = 0.822

Estimates of correlation coefficients between the different measures splitting the adult group by the eyeblink conditioning ISI, together with 95% CIs, and p-values.

## Discussion

Our study shows weak and uncertain correlations between the cerebellar-dependent sensorimotor tasks for both children and adults. Performance on all tasks improves with age – older children learn faster and show better precision in their responses. However, when controlling for age, other relationships between the tests are weak or absent. Figure 2 summarizes the strengths and directions of the correlations for all groups. All of the correlation estimates, except the negative correlation between prism deviation and percent CR in the child group, are less than 0.3 in strength – which is a small effect size according to Cohen’s convention^44^. When looking at the two adult groups separately, based on interstimulus interval (ISI) during eyeblink conditioning, a few correlations are estimated to be above 0.3. However, the direction of all correlations in this study are associated with a large uncertainty as each estimate’s 95% confidence interval (CI) crosses the zero mark (Fig. 2).

Although medium in effect size, the children’s negative correlation between prism deviation and CR percentage is uncertain. As we correlated many measures, it is expected that some measures will correlate by chance. Moreover, if we assume that there is something to this correlation, it would mean that less adaptation to the prisms is associated with more acquired CRs during eyeblink conditioning. This does at least not seem to capture any common aspect of cerebellar function. This conclusion is further reinforced by the fact that there is no association between prism adaptation and eyeblink conditioning in the adult group.

Further, the correlations with a medium effect size that were present only among one or the other adult group are also uncertain and somewhat difficult to interpret. Seemingly contradictory, the tapping variability correlates positively with CR percentage but negatively with CR onset in the ‘ISI 500’ group. Thus, more precise tapping is associated with less learning but also with more well-timed CRs during eyeblink conditioning. The positive correlation between prism adaptation and CR onset in the ‘ISI 300’ group makes more sense from a cerebellar learning perspective, i.e. that greater adaptation to prisms could be associated with better timed CRs. On the other hand, better spatial learning does not necessarily mean better temporal learning even though the cerebellum could play a general modulating role in various types of sensorimotor learning. Based on this study we cannot draw any conclusions from these potential relationships or regarding whether the degree of cerebellar contribution differs during different ISIs.

Even though the results in our sample indicate almost non-existent associations, we do not have enough data to prove that there is a complete lack of correlations. Rather, our findings suggest that if correlations exist, they are weak. We have, however, already deemed it implausible that the population level correlation would be exactly zero between the different performance measures. Performances on any pair of tasks are likely to correlate to some extent. However, the lack of somewhat stronger correlations after controlling for age, and the uncertainty of the relationships between all the measures, indicate that eyeblink conditioning, finger tapping, and prism adaptation do not capture any general cerebellar function and hence are not interchangeable. Rather, performance on these tasks appear to reflect separate cerebellar or extra-cerebellar mechanisms – with little, if any, overlap. This contradicts an earlier study in which finger tapping variability correlated negatively with CR percentage in eyeblink conditioning^23^. Our results may seem surprising given that the structure of the cerebellum is highly uniform, but recent evidence demonstrates that there are differences in the neurophysiological properties across the cerebellum^45^. Thus, one possible interpretation of our results is that eyeblink conditioning, finger tapping, and prism adaptation all depend on the cerebellum, but on different parts of the cerebellum, or that the underlying neural mechanisms are not the same.

One alternative explanation to our results is that common cerebellar functioning may not be clearly expressed with these methods and measures due to other cognitive processes that might disguise underlying relationships. The cerebellum is involved in various cognitive systems^16,46^ and lesions to the cerebellum can cause impairments of language, attention, and executive functions^16,47^. Recently, it was shown that human subjects can produce well-timed voluntary blink responses indistinguishable from other CRs^35^. It has also been suggested that associative learning based on eyeblink conditioning is a high-level cognitive process, and there is substantial evidence showing that awareness of the relationship between the CS and US influences the results^48–50^. In our study, we did not inform the subjects about the relationship between the stimuli. To distract them from the experiment, the subjects were instructed to pay attention to the film. Yet, we do not know if the subjects actually complied with these instructions, and we do not know to what extent watching the film blocked cognitive influences. As is often the case in studies on human eyeblink conditioning, the inter-individual variability was high^30^. Indeed, some individuals produced no CRs whatsoever. Others produced CRs after only a few paired trials. Perhaps some of the participants became aware of the relationship between the stimuli and blinked voluntarily so as to avoid the slightly unpleasant US. In prism adaptation, the target is known and the participants got feedback on the pointing errors in-between each trial. Several non-cerebellar factors may have influenced their performance. Some of the participants seemed to enjoy the task and laughed at the results, while others made more effort to reach the target and seemed embarrassed when they realised that their pointing was way off from where they aimed at.

It is unlikely that the substantial inter-individual variability in this and other studies is due entirely to differences in cerebellar function since severe cerebellar deficits would probably also result in other, more prominent, symptoms. Rather, we believe that the inter-individual variability reflects the fact that even simple behaviors, such as the ones we recorded here, are a product of genetics, environmental factors, and experiences during development such as relationships, stress, sensory stimulation, diet, intestinal flora, and hormones^51^. Ultimately, differences in these factors mean that individuals will differ in how they learn, think about, and behave during an experiment, resulting in large inter-individual variability. Cognitive processes are commonly characterized by high inter-individual variability^52^.

As we have reported earlier^30^, older children show more CRs, and more precisely timed CRs. In addition, the children show greater prism adaptation, less tapping variability, and faster RT, the older they are. That older children perform better than younger children in tasks like these could reflect that sensorimotor control improves during development. Compared to adolescents, children have been found to recalibrate slower after prism adaptation, which has been linked to an immature visuomotor system^53^. Compared to adults, children have been found to display a different brain activation pattern during finger tapping, perhaps because of less automatic tapping performance^54^. The cerebellum develops throughout adolescence^55^. Myelination of axons in the brain continues during development until late adolescence and is important for motor and sensory functions^52,56,57^. Further, development of cognitive functions is associated with maturity and myelination. Brain plasticity, which enables learning, peaks during sensitive periods during childhood. Although brain plasticity is associated with substantial inter-individual variability throughout life^52^, sensitive periods for the development of sensory processing occur early in childhood while cortical maturation continues throughout adolescence^58,59^. Moreover, the complexity of learning mechanisms seem to increase with maturation^58^. The adults in our study show more CRs, greater prism adaptation, less tapping variability, and faster RT, than the children. Our previous studies showed that children older than 9 years old can reach the level of performance as young adults in acquiring CRs during eyeblink conditioning with a 500 ms ISI, whereas children younger than 9 years old acquire considerably less CRs^30,31^.

## Conclusions

Our results show weak and unclear correlations between eyeblink conditioning, finger tapping, and prism adaptation. This suggests that the neural mechanisms underlying the performances do not substantially overlap, and that these three tasks measure different aspects of cerebellar function. Alternatively, the shared cerebellar contribution to these three tasks is small compared to the influence of other brain functions and factors. Our results highlight that one must be careful when using eyeblink conditioning, finger tapping, and prism adaptation to make inferences about general cerebellar function or dysfunction. However, in combination, these quantifiable and non-invasive behavioral tests may contribute to a nuanced picture of human cerebellar function during development. Further, before interpreting a poor performance on these tests as cerebellar dysfunction in groups with neurodevelopmental disorders, more research is needed to investigate how maturation affects the results in these disorders during development.

## Supplementary information

Supplementary Information.

## Data Availability

All data analyzed during this study are included in this published article and its Supplementary Dataset file.
