# Supplementary material for: Weak correlations between cerebellar tests
Source: Sci Rep. 2020 Jun 2;10:9003. doi: 10.1038/s41598-020-65886-1 (PMC7265407; doi:10.1038/s41598-020-65886-1)
Supplement: Supplementary file 1 — Supplementary Information. [file 41598_2020_65886_MOESM1_ESM.pdf]

Weak correlations between cerebellar tests.

Karolina Löwgren, Rasmus Bååth, and Anders Rasmussen.

ISI	sex	iqscore	agemonths	rtmedian	prodsd	prismsdev	perccr	onsetcr
500	1	100	124	261,0	38,9		0,18	292,8
500	1	120	126	266,5	43,1		0,75	345,9
500	2	115	126	238,5	27,3		0,1	241,8
500	1	125	132	224,5	42,9		0,14	419,6
500	2	115	117	263,0	49,0		0,08	455,3
500	1	115	88	550,5	50,1		0,14	245,6
500	2	120	108	265,5	35,6		0,41	350,0
500	2	125	117	284,5	45,7		0,34	312,0
500	1	90	108	233,5	39,9		0,29	404,0
500	2	80	90	349,0	54,5		0,05	332,5
500	2	95	127	227,5	36,9		0,55	317,9
500	1	120	126	403,5	38,6		0,18	378,6
500	1	115	133	248,0	29,9		0,81	377,1
500	1	105	126	237,0	30,4		0,43	321,6
500	1	90	90	275,0	32,5		0,11	245,3
500	2	120	108	292,0	36,9		0,21	341,4
500	2	100	104	279,5	48,3		0,03	436,0
500	2	95	97	305,0	42,8		0,05	178,5
500	2	125	121	354,5	47,1		0,08	247,3
500	2	115	84	307,5	46,4		0,2	219,0
500	2	90	126	275,0	39,5		0,53	357,4
500	1	100	132	301,5	37,0		0,38	407,9
500	1	125	126	349,5	34,1		0,79	341,2
500	2	105	87	234,5	52,2	-1	0,11	390,8
500	1	80	89	525,5	65,2	-0,2	0,11	274,0
500	2	100	84	396,5	64,0	4	0,03	139,0
500	1	100	81	385,0	51,7	0,6	0,42	334,3
500	2	115	83	429,0	78,2	1,8	0,03	268,0
500	2	90	90	380,5	46,3	1	0,06	324,5
500	1	90	88	381,0	54,2	0	0	
500	1	90	86	391,5	56,9	2	0,25	353,7
500	2	90	97	290,0	48,6	3,1	0,35	399,1
500	2	80	104	297,0	35,7	2,7	0,03	417,0
500	1	120	96	278,0	48,9	3	0,18	328,7
500	1	95	94	465,0	45,8	5	0	
500	1	90	104	306,0	47,0	3,4	0,66	317,5
500	1	80	113	284,0	40,2	8,7	0,03	415,0
500	1	100	108	406,5	59,1	4	0,24	328,0
500	1	110	109	298,0	35,6	2,4	0,79	338,3
500	2	125	111	412,5	32,0	1,8	0,35	341,1
500	1	90	108	296,5	49,4	2,9	0,76	330,2
500	2	140	83	513,0	42,4	1,4	0,03	355,0
500	1		310	186,0	20,3	3,8	0,79	304,5
500	1		671	199,5	27,4	0,5	0,44	316,4

500	2	325	182,0	17,4	3,4	0,47	345,7
500	2	397	204,0	13,7	8,0	0,03	427,0
500	2	285	177,0	19,0	3,0	0,23	291,8
500	2	247	172,5	14,0	3,6	0,49	337,7
500	2	268	219,5	20,2	3,0	0,23	266,0
500	1	320	183,0	21,7	1,6	0,58	315,3
500	1	291	187,0	26,5	3,3	0,44	326,4
500	2	263	175,5	20,1	3,5	0,38	368,6
500	1	290	186,5	21,6	1,1	0,26	350,0
500	1	532	164,5	22,8	3,0	0,77	275,0
500	1	424	171,0	24,1	7,0	0,81	321,5
500	2	307	172,0	16,5	2,0	0,08	374,7
500	1	273	177,0	19,8	3,2	0,08	297,0
500	1	259	183,5	23,4	3,5	0,97	203,6
500	1	341	188,5	17,6	2,8	0,79	289,2
500	1	300	191,0	34,7	2,9	0,89	274,9
500	1	380	157,0	32,8	4,3	0,57	322,7
500	1	336	244,0	13,2	0,2	0,81	305,6
500	1	327	194,0	25,2	2,6	0,78	347,9
500	1	528	171,0	23,0	7,7	0,32	254,6
500	1	298	258,5	22,2	5,4	0,38	297,6
500	1	262	185,5	22,9	3,0	0,34	314,8
500	2	489	186,0	11,4	2,5	0,25	373,6
300	1	343	193,5	13,9	8,7	0,00	
300	2	533	173,5	20,0	1,8	0,10	163,5
300	2	513	186,5	22,4	3,9	0,00	
300	2	240	186,0	26,9	3,9	0,47	196,8
300	1	259	233,5	25,5	3,2	0,62	225,2
300	2	342	198,0	23,7	4,8	0,26	235,1
300	2	257	184,0	26,0	4,3	0,10	187,3
300	2	391	210,5	41,5	4,2	0,18	219,5
300	2	260	177,0	21,0	4,5	0,21	224,5
300	1	310	212,5	23,7	2,6	0,64	184,2
300	2	288	157,5	20,4	5,2	0,60	227,9
300	1	226	188,5	16,7	5,9	0,00	
300	2	304	211,0	19,9	5,4	0,00	
300	2	262	190,5	17,3	6,9	0,58	241,2
300	1	421	190,0	32,4	2,5	0,47	224,8
300	1	256	176,0	17,5	5,1	0,69	231,1
300	1	266	217,5	34,3	6,3	0,71	207,1
300	1	248	174,5	24,2	1,7	0,11	231,5
300	1	288	208,5	33,2	3,7	0,90	203,1
